# Two-dimensional lithium diffusion behavior and probable hybrid phase transformation kinetics in olivine lithium iron phosphate

**DOI:** 10.1038/s41467-017-01315-8

**Published:** 2017-10-30

**Authors:** Liang Hong, Linsen Li, Yuchen-Karen Chen-Wiegart, Jiajun Wang, Kai Xiang, Liyang Gan, Wenjie Li, Fei Meng, Fan Wang, Jun Wang, Yet-Ming Chiang, Song Jin, Ming Tang

**Affiliations:** 1 Department of Materials Science & NanoEngineering, Rice University, Houston, TX 77005 USA; 2 Department of Chemistry, University of Wisconsin-Madison, Madison, WI 53706 USA; 3 Department of Materials Science & Engineering, Massachusetts Institute of Technology, Cambridge, MA 02139 USA; 4 Photon Science Division, Brookhaven National Laboratory, Upton, NY 11973 USA

## Abstract

Olivine lithium iron phosphate is a technologically important electrode material for lithium-ion batteries and a model system for studying electrochemically driven phase transformations. Despite extensive studies, many aspects of the phase transformation and lithium transport in this material are still not well understood. Here we combine *operando* hard X-ray spectroscopic imaging and phase-field modeling to elucidate the delithiation dynamics of single-crystal lithium iron phosphate microrods with long-axis along the [010] direction. Lithium diffusivity is found to be two-dimensional in microsized particles containing ~3% lithium-iron anti-site defects. Our study provides direct evidence for the previously predicted surface reaction-limited phase-boundary migration mechanism and the potential operation of a hybrid mode of phase growth, in which phase-boundary movement is controlled by surface reaction or lithium diffusion in different crystallographic directions. These findings uncover the rich phase-transformation behaviors in lithium iron phosphate and intercalation compounds in general and can help guide the design of better electrodes.

## Abstract (web summary)

Lithium transport and phase transformation kinetics in olivine LiFePO_4_ electrode remain not fully understood. Here the authors show that microsized olivine particles possess 2D lithium diffusivity and exhibit a possible hybrid mode of phase boundary migration upon cycling.

## Introduction

Advancement in electrochemical energy storage technology has seen the development of many important lithium-ion battery electrode materials that undergo electrochemically driven first-order phase transformations, exemplified by the graphite anode and olivine lithium iron phosphate (LiFePO_4_) cathode^1–5^. Recent theoretical^6–8^ and experimental^9–12^ studies unveil the formation of metastable solid solution LiFePO_4_ at high (dis)charge rates. Nevertheless, the two-phase coexistence behavior is still expected to dominate at relative low (dis)charge rates and in larger, micron-sized particles, where phase-boundary movement has an important role in the (dis)charge kinetics. However, the morphology and migration behavior of phase boundaries in LiFePO_4_ (LFP) continues to be a subject of dispute despite numerous experimental^13–18^ and modeling^8, 19–24^ efforts to seek clarification.

When first-order transformations involve two phases with different compositions, the growth of new phase is conventionally categorized as being bulk diffusion limited (BDL) or interface-source limited^25^. The rate of phase-boundary migration is controlled by the rate at which species are transported to the boundary via long-range diffusion in BDL growth, or the rate atoms cross the boundary in interface-source-limited growth. For ion-insertion materials, however, the fact that they are open systems (i.e., exchanging mass with environment) may give rise to an entirely new type of transformation kinetics, in which the phase-boundary movement is controlled by how fast ions are inserted or extracted across the electrode/electrolyte interface. This surface reaction-limited (SRL) phase-boundary migration mechanism is first postulated by Singh, Ceder, and Bazant (SCB)^19^ to explain (100)-oriented phase boundaries observed in partially delithiated LFP particles^13^. They argue that Li diffusion in LFP is sufficiently facile so that surface reaction should be the rate-limiting step for phase growth. In the predicted SRL kinetics, phase boundary moves orthogonally to the Li surface flux direction at a constant velocity. However, although the SCB prediction is theoretically appealing and supported by indirect evidence, direct experimental confirmation of SRL phase-boundary movement has not been reported for any intercalation compounds so far. Although the ex situ observation of phase boundaries perpendicular to (010) particle surface in partially delithiated LFP platelet particles^13, 17, 26^ has been suggested as evidence for SRL boundary migration, it is recognized that such morphology is likely formed during phase-separation process under the influence of elastic strain energy^26^. Instead, existing in situ observations of microsized LFP particles upon (de)lithiation appear to only lend support to the BDL phase-growth mechanism^16, 27^. Whether phase transformations can operate in SRL mode under realistic electrochemical conditions is not only a scientifically significant question but also has practical implications for improving the performance of ion-insertion battery materials.

The transformation between FePO_4_ and LFP phases is intimately influenced by Li-diffusion behavior in LFP. Since the first-principles calculations by Morgan et al^28^. reported that Li diffusion in olivine structure is mainly confined to [010]-oriented open channels, the one-dimensional (1D) Li diffusivity has been widely regarded as a quintessential property of LFP. However, Amin et al.^29^ made a remarkable finding that Li has equal mobility along *b* and *c* axes in millimeter-sized LFP single crystals containing 2.5–3% Li–Fe anti-site defects through impedance measurement. Subsequently, Malik et al.^30^ predicted that anti-site defects can not only block the [010] diffusion channels but also decrease the energy barrier for inter-channel Li hopping, effectively reducing Li-diffusion anisotropy. Practically synthesized olivine cathodes typically contain non-negligible amount of anti-site point defects due to non-equilibrium synthesis conditions. Although these pioneering studies reveal the marked impact defects can have on Li transport in LFP, the single-crystal LFP examined in ref. ^29^ was grown by the floating zone method and differs from most LFP particles employed in batteries, which are primarily prepared by hydrothermal synthesis or solid-state reaction. Measurement of Li-diffusion anisotropy in LFP micro-particles and nanoparticles made by these methods is rare; it is not clear whether the 2D Li transport behavior of single crystals reported in ref. ^29^ is representative of all forms of LFP regardless of their synthesis methods.

To elucidate the phase transformation and ion diffusion characteristics in electrode materials like LFP, direct observations of the (de)lithiation process in individual particles are immensely valuable. Previous studies using in situ transmission electron microscopy (TEM)^16^, ex situ^17, 31, 32^ or in situ soft X-ray scanning transmission X-ray microscopy (STXM)^26, 33^, in situ/*operando* hard X-ray TXM in combination with X-ray absorption near-edge structure spectroscopy (TXM-XANES)^34–36^, and soft X-ray ptychographic microscopy have shed invaluable insights on phase-boundary orientation and movement^16, 18, 26, 35, 36^, reaction inhomogeneity^26, 31–35^, and (de)lithiation-induced mechanical strain^17, 18^ in LFP. In particular, TXM-XANES is a unique technique with large field-of-view (up to 40 × 40 μm) and spatial resolution down to nanoscale (~25 nm) that can be used to study composite electrodes (active material/carbon/binder) using a simple *operando* electrochemical cell^37^.

In this work, we use *operando* TXM-XANES imaging to track the electrochemical delithiation process in a composite electrode consisting of carbon black, binder, and single-crystal LFP microrods that are specifically synthesized to have their long-axis grown along the [010] direction. Interpreted by theoretical analysis and phase-field simulation, our experiment provides important insights into the Li-diffusion anisotropy and phase-boundary migration mechanisms in LFP and beyond. First, we confirm that hydrothermally synthesized microsized LFP particles may also have comparable Li-diffusion constants along the [010] and non-[010] directions, supporting that defect-induced (transversely) isotropic Li diffusivity is a ubiquitous feature of LFP and independent of synthesis method. A direct consequence of such behavior is that, against common belief, the (100) or (001) surfaces of LFP particles should be practically considered as active in the Li (de)intercalation process. Second, we are able to obtain the first direct proof of the previously predicted SRL transformation behavior. Moreover, our study reveals a more subtle and complete picture of phase transformations in LFP and intercalation compounds in general, as we show that the growth of new phase during (de)lithiation could potentially proceed through a hybrid mode, in which phase-boundary movement follows SRL and BDL kinetics along different directions, respectively.

## Results

### Synthesis and characterization of LiFePO_4_ microrods

**Fig. 1:**
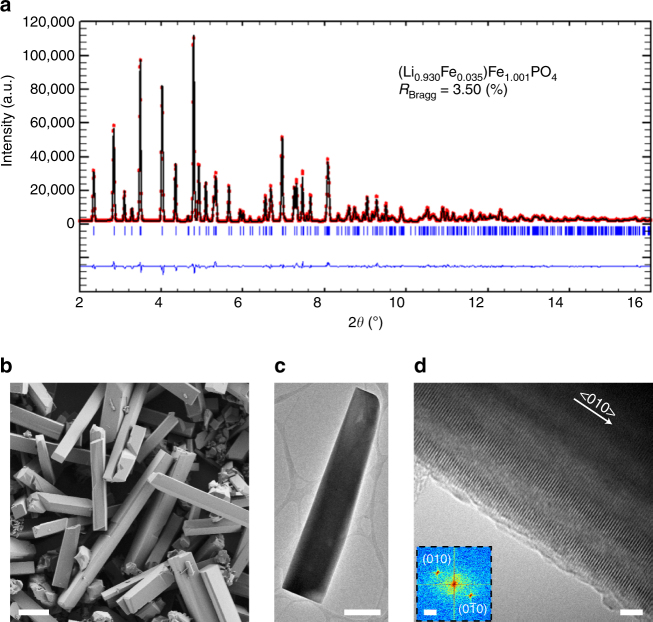
Structural characterizations of the LiFePO_4_ microrod sample. **a** High-resolution PXRD of the LFP microrods and the Rietveld-refined pattern using the Fe-rich model (*λ* = 0.2128 Å). **b** SEM image of the LiFePO_4_ microrods. The scale bar is 10 μm. **c** Low-magnification TEM image of a single LiFePO_4_ microrod. The scale bar is 1 μm. **d** High-resolution TEM image of a representative LiFePO_4_ microrod, showing that the microrod is a single crystal with the long-axis direction along <010>. Inset is the corresponding FFT confirming that the observed lattice planes are (010) family of planes. The scale bars are 5 nm and 2 nm^−1^, respectively

We first synthesized the LFP microrods via a hydrothermal reaction using LiOH, FeSO_4_·7H_2_O, and NH_4_H_2_PO_4_ as the precursors for Li, Fe, and P, respectively (see synthetic details in “Methods”). Nitrilotriacetic acid (NTA) was used as a complexing reagent to Fe^2+^ to avoid the formation of iron hydroxide and maintain low supersaturation to favor anisotropic crystal growth^38^. No microrods could be made without NTA. The phase identity of the as-synthesized sample was confirmed as orthorhombic LFP (space group *Pmna*) by synchrotron-based powder X-ray diffraction (PXRD, Fig. 1a). Chemical analysis using inductively coupled plasma atomic emission spectrometry (ICP-AES) revealed an Fe-rich sample with average Fe:Li ratio of 1.11:1. Rietveld refinement was then performed using an Fe-rich model, which yields a structural formula of Li_0.93_Fe_0.035_Fe_1.001_PO_4_ and a Bragg factor of 3.50%. The anti-site concentration is hence estimated to be 3.5% (i.e., lithium octahedral M1 site 4a contains 3.5% iron). This result is comparable to the anti-site defect concentration (2.5–3%) found in LFP single crystals grown by optical floating zone technique^29^ and slightly lower than those previously reported for LFP samples prepared via low-temperature syntheses (5–10% cation disorder)^39, 40^. The PXRD pattern was also refined under the assumption of no anti-site defects, which yields less satisfactory agreement with a larger Bragg factor of 4.93% (Supplementary Fig. 1). Scanning electron microscopy (SEM) shows that these LFP products are well-faceted microrods of 2–7 μm in thickness/height and tens of micrometers in length (Fig. 1b). Many of them have a rectangular or nearly square cross-section (Fig. 1b, c). Notably, high-resolution TEM and the corresponding fast-Fourier-transform (FFT) analysis of more than 15 microrods consistently confirm that the microrods are single crystals and their long-axis direction is along [010] (Fig. 1d). Because the lithium-ion channels in LFP are also along the [010] direction, these microrods provide an excellent and unique platform to study the electrochemical delithiation reaction in LFP.

### *Operando* X-ray spectroscopic imaging of delithiation in LiFePO_4_

**Fig. 2:**
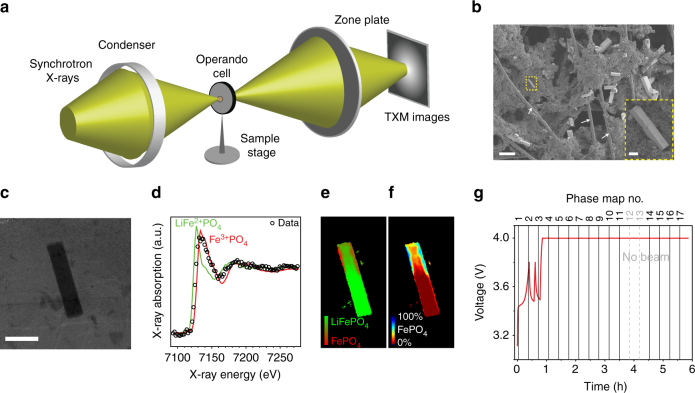
*Operando* TXM-XANES experiments on LiFePO_4_ electrode. **a** Schematic illustration of the TXM-XANES setup. **b** SEM image of the LiFePO_4_ electrode used for the *operando* experiments, which is prepared by depositing LiFePO_4_ microrods, carbon black, and PVDF binder onto a carbon paper made of carbon microfibers (indicated by the white arrows). The scale bar is 40 μm. Inset is a high-magnification view of a single LiFePO_4_ microrod. The scale bar is 5 μm. **c** 40 × 40 μm (256 × 256 pixels) *operando* TXM view of the LiFePO_4_ electrode, showing a single LiFePO_4_ microrod in the presence of carbon black, PVDF binder, and liquid electrolyte. Scale bar is 10 μm. **d** XANES spectrum collected from the *operando* experiment (black open circle) in comparison with those of pristine LiFe^2+^PO_4_ (green curve) and Fe^3+^PO_4_ (red curve). XANES spectrum at each pixel of the TXM images is fitted by a linear combination of the LiFe^2+^PO_4_ and Fe^3+^PO_4_ reference spectra to determine the ratio between the two phases, from which the two-phase chemical map shown in **e** can be constructed. **f** Single-phase chemical map derived from the two-phase chemical map. The “jet” color-scale corresponds to the fraction of the FP phase. **g** Voltage vs. time profile of the *operando* delithiation experiment, which consists of constant-current, OCV, and constant-voltage steps. The cell was charged at a constant rate of 1/5 *C* with two intermittent OCV periods and then held at a constant voltage of 4 V. TXM images were collected with a time interval of ~0.35 h without interrupting the delithiation process to produce chemical-phase maps

We used the *operando* TXM-XANES technique to visualize the electrochemical delithiation of the LFP microrods. Figure 2a is a schematic illustration of the experimental setup. Synchrotron hard X-rays pass through a perforated 2032-type coin-cell containing a composite LFP electrode consisting of LFP particles, carbon black, and polyvinylidene fluoride (PVDF) binder coated on a carbon microfiber paper (Fig. 2b) and other battery components such as Li metal counter electrode, liquid electrolyte, and separator. The cell was sealed by two layers of Kapton tape on both sides of the cell cases, which allow X-ray transmission but keep the cell from exposing to air. To perform chemical-phase mapping, the energy of the incident X-ray is scanned across the Fe *K*-edge (7112 eV) at a step size of 2 eV and a series of absorption-contrast TXM images (40 × 40 μm, 256 × 256 pixels) are collected, one image at each energy (one example shown in Fig. 2c). XANES spectrum at each pixel of the TXM images is fitted by a linear combination of the LiFe^2+^PO_4_ and Fe^3+^PO_4_ (FP) reference spectra to determine the ratio between the two phases (Fig. 2d) so that red (100% FP) and green (100% LFP) colors can be accordingly assigned to generate the two-phase chemical map (Fig. 2e). Using the information contained in the two-phase map, single-phase chemical map can also be produced and is best shown in the form of “jet” color-map (Fig. 2f), in which the color-scale represents the spectral fraction of the FP phase (red, 0%; blue, 100%). The *operando* experiment consists of constant-current, open-circuit-voltage (OCV), and constant-voltage steps (Fig. 2g). The reason to have OCV periods is to investigate what happens to the LFP particle during relaxation. The *operando* cell was first charged (delithiated) to 3.8 V at a rate of 1/5 *C* (1 *C* = 170 mA g^−1^) and allowed to rest under OCV for 10 min. Afterwards, the cell was charged at 1/5 *C* rate again and delithiation resumed for a very short duration, the cell was put to a second rest for 10 min when its voltage reached 3.8 V again. Subsequently, it was charged to 4 V at 1/5 *C* rate and held under constant voltage for the remainder of the experiment. To make sure that the cell would function normally, we routinely cycled the cell under potentiostatic mode between open-circuit voltages to 4 V for one cycle before the *operando* experiments.

**Fig. 3:**
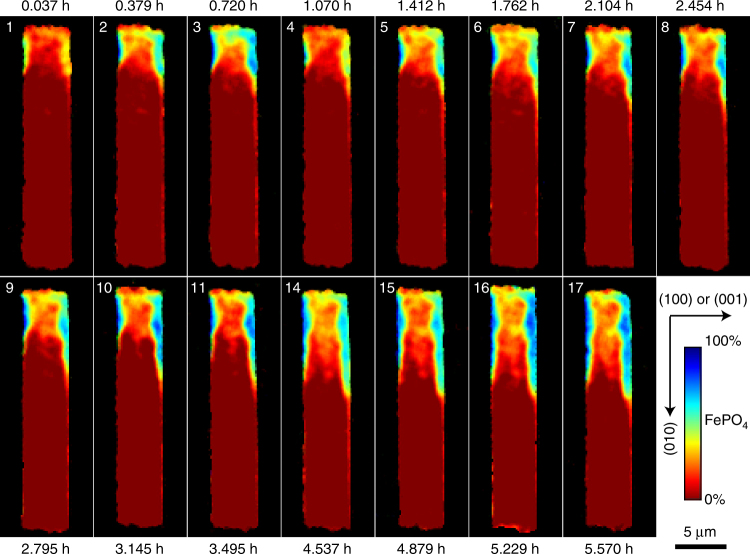
*Operando* visualization of delithiation of a single-crystal LiFePO_4_ microrod. 2D depth-averaged FePO_4_ single-phase chemical maps (58 × 162 pixels; 1 pixel corresponds to ~160 nm) taken at different states of charge show the evolution of the phase state of a LiFePO_4_ microrod along different crystallographic directions. The long-axis of the LiFePO_4_ microrod is along [010] and its short-axis is along [100] or [001]. The “jet” color-scale corresponds to the fraction of the FP phase (red, 0% FePO_4_; blue, 100% FePO_4_). All phase maps share the same 5 μm scale bar. Note that phase map 12 and 13 were not collected because X-ray beam was not available due to synchrotron refill

TXM images of a single LFP microrod (~25 μm in length and 5 μm in width) were collected at multiple states of charge with a time interval of ~0.35 h to generate single-phase maps of the FP phase, which shows the evolution of the electrochemical delithiation process (Fig. 3, map 1–17). The same chemical maps are also shown in a time sequence in Supplementary Movie 1. This microrod was chosen because it was not overlapping with any other LFP particles and produce high-quality images. After collecting each set of TXM data, the area of study was allowed to rest (not exposed to X-rays) for ~14 min to minimize any potential damage to the *operando* cell induced by the X-ray beam. We note that hard X-rays are strongly penetrating so that other battery components, such as Li electrode, carbon black, binder, organic electrolyte, and separator, do not have enough contrast to be visible. Significantly, as the crystallographic orientation of the LFP microrod is separately determined from TEM characterization (Fig. 1d), we can analyze the orientation dependence of the delithiation process in LFP particles in a way that is not attainable in previous in situ or *operando* studies^33–35^. We do caution that even though the long-axis of the LFP microrod under observation is clearly along [010], TXM-XANES cannot distinguish whether the crystallographic direction of its short-axis in the 2D phase maps is [100] or [001]. This ambiguity, however, does not affect the discussion on the Li diffusion and phase-transformation mechanisms presented below. From now on, we refer to this short-particle dimension in the phase maps as the [100]/[001] axis.

The first striking observation from the TXM single-phase maps is that delithiation initiated on (100)/(001) instead of (010) particle surfaces. Shortly after charging started, two FP domains appeared and then grew on the opposite (100)/(001) surfaces (Fig. 3, phase map 1–3). In comparison, delithiation on (010) surface proceeded much more slowly. When examining the LFP electrode used in the *operando* cell under SEM, we found that LFP particles in the electrode are not uniformly covered by carbon black because of their large size (Fig. 2b and inset). It is likely that the (010) surfaces of this particular particle observed by TXM have poor contact with the conductive network, making them relatively inactive for Li intercalation. On the other hand, the locations on (100)/(001) surfaces where FP phase first nucleated may have the best electrical contact with carbon black. Although this hypothesis could not be verified by TXM because carbon is transparent to hard X-rays, it is indeed consistent with the SEM observation (Fig. 2b inset). Interestingly, the FP phase region appears to be re-lithiated during the OCV period (phase map 3 and 4), which may be caused by inter-particle Li exchange. When the charging process resumed, FP phase re-appeared at the original nucleation sites on (100)/(001) surfaces (phase map 5), suggesting that the FP phase did not completely vanish during rest and grew again when the particle was delithiated again. We also observed spatial variation in the concentration profile within each FP region in the chemical maps. A possible cause is the non-uniform delithiation in the direction in parallel with the X-ray beam (i.e., variation is in the depth direction), which can result from variation in the electrical contact between the (100)/(001) particle surfaces and the uneven carbon conductive network as observed in the *operando* electrode (e.g., Fig. 2b). In addition, the inhomogeneity in delithiation can be further enhanced by the asymmetrical composition dependence of the surface reaction rate constant as revealed by recent experiment and theory^26, 41^. After integration of Fe^3+^PO_4_ X-ray absorption signal in the beam direction, the non-uniform delithiation could be present as “spatial variation” in the depth-averaged 2D chemical maps shown in Fig. 3. The coherency stress arising from the lattice misfit between LFP and FP may also contribute to the spatial fluctuation in TXM images by triggering the morphological instability of the delithiation front^33^. However, the effect of misfit stress is likely to be limited in our sample as it is expected to be effectively relaxed by defects such as dislocations and cracks in microsized LFP particles, which will be discussed in more details below.

**Fig. 4:**
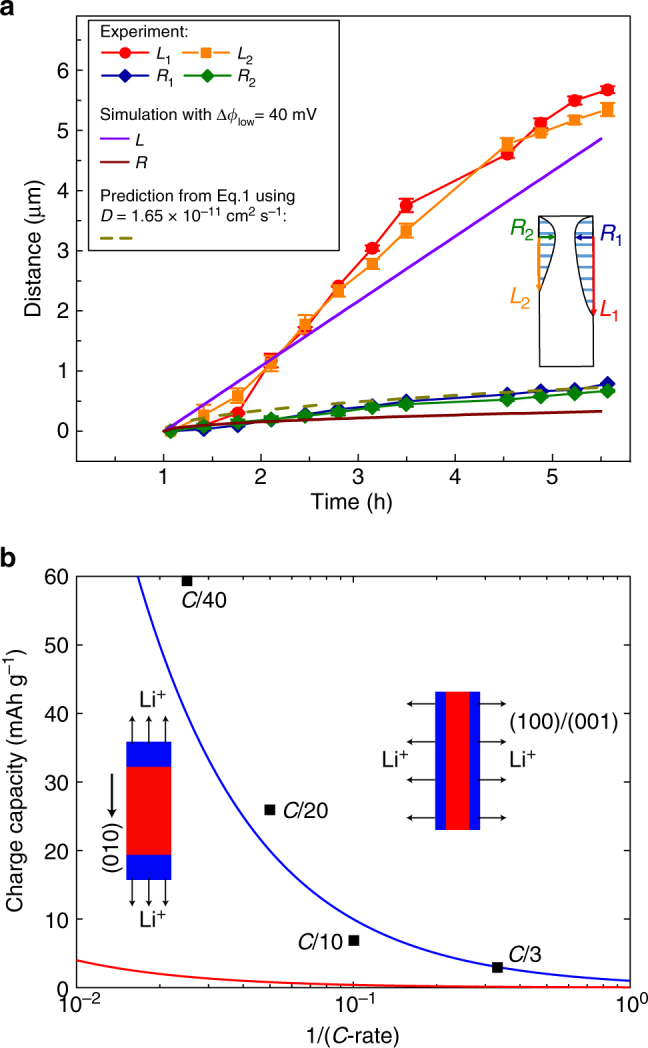
Experiment-modeling comparison of phase growth and charging kinetics. **a** Time evolution of [100]/[001] (*R*_1_ and *R*_2_) and [010] (*L*_1_ and *L*_2_) dimensions of the two FePO_4_ domains in the LiFePO_4_ particle during delithiation, compared against prediction from Eq. 1 with *D* = 1.65 × 10^−11^ cm^2 ^s^−1^ (dashed line) and phase-field simulation (solid lines) with *ϕ*_low_ = 40 mV (see text). The inset schematically shows how *R* and *L* are measured. The measurements are performed on phase maps 4–17 (starting at *t = *1.070 h). *R* and *L* represent the width and length of the FePO_4_ region at its widest point (from the two nucleation sites near the two top corners of the microrod). **b** Galvanostatic charging capacity of LiFePO_4_ electrode at different *C*-rates vs. predictions from a simple delithiation model assuming *D* = 10^−11^ cm^2^ s^−1^ and that the delithiation front propagates along [100]/[001] (blue line) or [010] (red line).

The observed FP phase growth suggests that Li was extracted from (100)/(001) surfaces during delithiation. This would not be possible if Li can only move along [010] channels. Our LFP sample thus must have a non-negligible diffusion constant in the [100]/[001] direction, which is made possible by anti-site lattice defects present in the particles. To estimate *D*_[100]/[001]_ in the observed LFP particle, we measured from the single-phase chemical maps the [100]/[001] dimensions of the two FP domains (denoted as *R*) against time after charging was resumed at ~1 h since the experiment commenced. As shown in Fig. 4a inset, *R* is measured at the nucleation sites of the two FP domains, which are defined as the pixels with the largest FP phase fraction in phase maps 4–17 of Fig. 3. We chose 10% as the minimum FP phase fraction to consider that a pixel belongs to a FP domain. As the chemical information contained in each pixel of the 2D maps is averaged along the X-ray beam direction (or the particle depth direction), a pixel with 10% FP phase fraction likely means that the top or bottom surface of the LFP particle has already been fully delithiated. In Fig. 4a, *R* represents the width of the FP region at its widest point. The measured *R*(*t*) was then compared with the theoretical prediction assuming that FP phase growth along [100]/[001] is diffusion-controlled (Fig. 4a). In literature, the relation *X *~ (*Dt*)^1/2^ is typically used to estimate the location of Li-diffusion front *X* in intercalation compounds as a function of time *t*. It should be pointed out that this expression applies to single-phase-diffusion process but is not proper for predicting diffusion-controlled phase-boundary migration, which in the limit of small Li solubility is described by (see Supplementary Information):

1 $$X\sim \sqrt {\frac{{c_{{\rm{FP}}}^{{\rm{eq}}} - {c^{{\rm{surf}}}}}}{{c_{{\rm{LFP}}}^{{\rm{eq}}} - c_{{\rm{FP}}}^{{\rm{eq}}}}}2Dt} ,$$

where $c_{{\rm{FP}}}^{{\rm{eq}}}$ and $c_{{\rm{LFP}}}^{{\rm{eq}}}$ are the Li concentrations of FP and LFP phases at two-phase equilibrium, respectively, and *c* ^surf^ ≈ 0 is the Li concentration at particle surface during delithiation. Equation 1 shows that using *X *~ (*Dt*)^1/2^ will overestimate the phase-boundary velocity especially when Li has limited solubility in both phases, i.e., $c_{{\rm{FP}}}^{{\rm{eq}}} \ll 1$ and $1 - c_{{\rm{LFP}}}^{{\rm{eq}}} \ll 1$. The equilibrium solubility of lithium in FP and LFP phases is found to be strongly particle size-dependent. Although the solubility shows significant increase with reducing particle size below 100 nm^42, 43^, the $c_{{\rm{FP}}}^{{\rm{eq}}}$ ($c_{{\rm{LFP}}}^{{\rm{eq}}}$) value for larger sub-micron particles reported in literature is in the range of 1–2% (98–99%)^42, 43^, with even lower value possible in microsized LFP particles. Several recent *operando* synchrotron X-ray diffraction or spectroscopic studies provide evidence that the solid solution can be dynamically extended and even bypass the first-order phase transformation in LFP at high (dis)charge rates^11, 12, 26, 44^. However, such observations are invariably obtained from nanoscale LFP, and the phenomenon is not found in similar experiments on microsized particles^45, 46^. Therefore, the large microsized LFP particles examined in this study should have a very limited range of solid solution under both equilibrium and non-equilibrium conditions. Here we use the solubility values reported by Meethong et al.^42^ for a 113 nm LFP sample, $c_{{\rm{FP}}}^{{\rm{eq}}}$ = 0.01 and $c_{{\rm{LFP}}}^{{\rm{eq}}}$ = 0.99, which can be viewed as a lower bound estimate of the miscibility gap for our sample. As shown in Fig. 4a, good agreement between Eq. 1 and the experimental curve is obtained when the [100]/[001] Li-diffusion constant *D*_[100]/[001]_ = 1.65 × 10^−11 ^cm^2^ s^−1^ is used in the prediction.

In an independent experiment, the Li-diffusion constant of the sample was measured in the single-phase region by galvanostatic intermittent titration technique (GITT). The obtained diffusivity value of 0.70 × 10^−11 ^cm^2 ^s^−1^ (measured at *x = *0.01 for Li_1−*x*_FePO_4_) is very close to *D*_[100]/[001]_ estimated from TXM images, which is smaller than the theoretically predicted 10^–8^–10^−7 ^cm^2 ^s^−1^ for defect-free LFP^28^ but comparable to most experimentally measured values^29, 47–51^. Although we were not able to fully delithiate the microrod particles to measure the value of *D* in FP-solid solution, most of the reported Li-diffusivity measurements in both LFP and FP single-phase regions show them to be comparable. As GITT is supposed to probe the largest component of the diffusion constant tensor in LFP, an important implication of the above results is that Li diffusivity along [100]/[001] is comparable to or even larger than the diffusion constant along [010]. The presence of relatively facile Li transport along [100]/[001] is further corroborated by galvanostatic charging of our samples at various *C*-rates (Fig. 4b). We find that the measured galvanostatic capacity could be well explained by a simple (dis)charge model (Supplementary Eq. 7 in Supplementary Information) when delithiation is assumed to proceed mainly along [100]/[001]. However, the predicted capacity will be one order of magnitude smaller than the experimental values if Li diffusion is assumed to occur mainly along [010]. Because this model only considers solid-diffusion resistance and omits other types of polarization (e.g., contact resistance), which will further reduce capacity, the predictions only represent an upper-bound estimate. Therefore, our observations provide convincing evidence that Li has similar diffusion constants between [100]/[001] and [010] directions and (100)/(001) surfaces can actively intercalate Li in LFP particles.

Further support to this conclusion is provided in Supplementary Fig. 2, which presents another set of *operando* TXM images of a microrod sample that was synthesized by similar method but has smaller particle sizes. The images clearly show the nucleation of FP phase at the center of the (100)/(001) side surfaces of the microrod, where particle is likely in good electronic contact with carbon black. Such phase-growth morphology is not possible without significant Li diffusion along [100]/[001]. This is the first time that 2D Li-diffusivity behavior is reported for LFP particles prepared by the widely used hydrothermal approach. Our finding verifies that the effect of lattice defects on reducing Li-diffusivity anisotropy in LFP is a universal phenomenon regardless of synthesis techniques. It also confirms the direct correlation between anti-site defects and Li-diffusion anisotropy as our sample and the previously reported millimeter-sized LFP single crystal^29^ have comparable anti-site concentrations of ~3%.

As the Li diffusivity of our sample is in the range of typical measured values for LFP in the literature, it may be inferred that most practically synthesized LFP contain sufficient amount of anti-site defects to induce the 2D Li-diffusion behavior. This raises the question on whether the intrinsic 1D Li-migration behavior of defect-free LFP crystal is relevant to practical applications. Accordingly, our thinking towards how olivine particles should be optimized to improve the rate capability of batteries may need to be changed. For instance, non-(010) surfaces should also be considered as active for Li intercalation. It thus may not be necessary to maximize (010) surface area and minimize [010] dimension of LFP particles to achieve optimal rate performance; reducing [100] or [001] particle dimension could also be beneficial. This indeed has been demonstrated in a recent study^52^, which finds that a [100]-oriented LFP nanoflake sample of ~12 nm thick in the [100] direction exhibits much a larger capacity at high rates than nanoparticles with a much smaller [010] dimension but increased [100] size.

We note that the lithium diffusivity under consideration here should be interpreted as ambipolar Li diffusivity, which incorporates the effect of ion–electron coupling on Li migration. With the local electroneutrality requirement, ambipolar diffusivity is commonly expressed as:

2 $$D = {D_{{\rm{L}}{{\rm{i}}^ + }}}{D_{{e^ - }}}{\rm{/}}({D_{{\rm{L}}{{\rm{i}}^ + }}} + {D_{{e^ - }}}),$$

where ${D_{{\rm{L}}{{\rm{i}}^ + }}}$ and ${D_{{e^ - }}}$ are the diffusion coefficients of Li^+^ and *e* ^−^, respectively. Electrical transport in LFP proceeds through the hopping of small polarons^53^. However, the role of electronic conduction in determining the ambipolar diffusivity in microsized LFP is still not well understood. The electrochemical impedance measurement by Amin et al.^29^ finds the electronic conductivity to be several orders of magnitude larger than ionic conductivity in millimeter-sized LFP single crystals. Nevertheless, the recent *operando* scanning TXM observation by Ohmer et al.^33^ provides evidence that electronic conduction could be the rate-limiting step upon charging of a LFP sample of comparable dimensions. Furthermore, Eq. 2 makes the assumption that the migration of cations and anions is coupled through electrostatic interaction only. As pointed out by Malik et al.^54^, this assumption may not be applicable to LFP as a strong binding energy and correlation in migration paths between polaron and Li ion are predicted from first-principles calculations^53, 55^, which supports the picture that Li^+^ and *e* ^−^ co-migrate as a neutral complex in LFP. In this scenario, *D* represents the diffusivity of Li^+^–*e* ^−^ complex and can no longer be reliably estimated from separately measured ionic and electronic conductivity, similar to the trapping effect discussed in literature^56, 57^. The implications of our observation for individual electron and lithium-ion conductivity are subject to further clarification with an improved understanding of the nature of the Li^+^–*e* ^−^ coupling in LFP.

In light of the finding above, another remarkable feature displayed by the chemical mapping data is that phase transformation proceeds along [010] at a much faster rate than [100]/[001] in the imaged microrod (maps 4–17, Fig. 3). Figure 4a shows the measured [010] FP-domain size *L*(*t*), defined as the distance between the nucleation site and the triple-phase junction (TPJ) on (100)/(001) surface, where FP, LFP, and electrolyte meet. If phase-boundary movement along [010] is also diffusion-limited, then *L*(*t*) ≈ *R*(*t*) should be expected given the 2D Li-diffusion characteristics, which nonetheless substantially underestimates *L*(*t*). To match the prediction by Eq. 1 to the measured *L*(*t*) would require *D*_[010]_ ≈ 10^−9 ^cm^2 ^s^−1^, which is two orders of magnitude larger than the experimental result and therefore not possible. However, the large [010] growth rate of FP phase can be explained if the advancement of phase transformation front along [010] is not controlled by bulk diffusion but surface reaction that regulates how fast Li can be extracted from (100)/(001) surfaces, in line with the predicted SRL kinetics. To shed more light on this hypothesis, a phase-field model^20^ is employed to simulate the delithiation process (see model details in “Methods”).

### Phase-field simulation of delithiation in LiFePO_4_

**Fig. 5:**
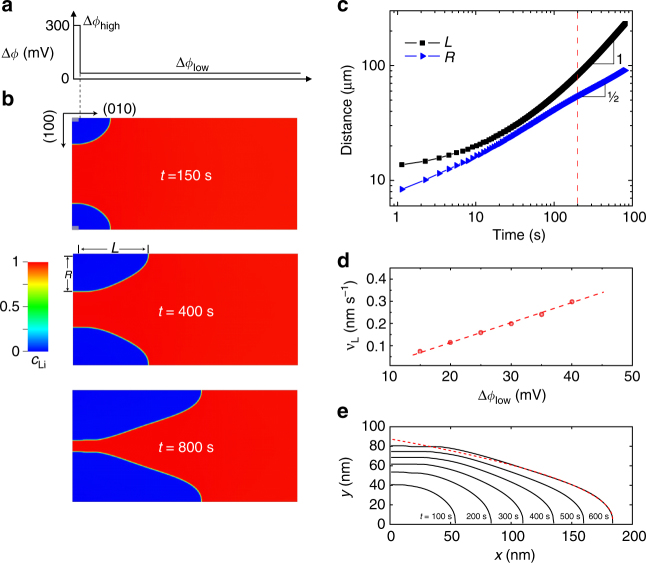
Phase-field simulation of delithiation in LiFePO_4_. **a** Illustration of stepwise overpotential applied on (100)/(001) surface. **b** Snapshots of FP phase morphology (blue domains) at time 150 s, 400 s, and 800 s in delithiation simulation with *ϕ*_low_ = 35 mV. **c** Log–log plot of the dimensions of FP domain along [010] and [100]/[001], defined by *L* and *R*, respectively, against delithiation time. *L*(*t*) and *R*(*t*) has a slope of 1 and ½, respectively. **d** TPJ velocity (circles) obtained from phase-field simulation as a function of *ϕ*_low_. The dashed line is a guide to eyes. **e** Time evolution of phase-boundary morphology. Phase boundary at different delithiation times exhibits steady-state morphology near TPJ (or [010] growth front), which can be described by a parabolic curve (red dashed line) *y *∝ $\sqrt {L\left( t \right) - x}$

We carried out delithiation simulation of a microrod in 2D to compare with the depth-averaged chemical maps from the experiment. Consistent with the TXM-XANES observation, the two (010) surfaces are assumed to be inactive in Li deintercalation in simulation by imposing a no flux boundary condition. Li is thus only allowed to be extracted from the (100)/(001) boundaries of the computation domain. As illustrated in Fig. 5a, stepwise overpotential is imposed on the two (100)/(001) side faces of the particle. A small section near the particle corner at *x = *0 is assigned a large overpotential Δ*ϕ*_high_ = 300 mV to represent the surface area in contact with carbon black where FP phase will first nucleate. Using even larger Δ*ϕ*_high_ has little effect on simulation results but makes calculation more difficult to converge. Owing to the poor electrical contact, the rest of (100)/(001) surfaces experience a lower overpotential at Δ*ϕ*_low_. As no spontaneous nucleation of FP phase was observed in the carbon-free surface region in the experiment, we set Δ*ϕ*_low_ below the transformation barrier or spinodal point of LFP phase, which is 45 mV in our model. A 2D Li diffusivity of *D* = 10^−11 ^cm^2 ^s^−1^ is chosen in accordance with experiment, and the exchange current density is set at 0.1 A m^−2^, comparable to the value recently reported in ref. ^32^. An incoherent phase boundary is assumed in the simulation, and therefore no misfit stress is present during phase transformation. Coherency stress rising from the lattice mismatch between FP and LFP phases can profoundly influence the phase-transformation behavior and Li transport in LFP by suppressing phase separation^8^, triggering interface instability^58^, and modifying the migration-energy barrier of Li^33, 59^. However, although nanoscale LFP is able to sustain the large coherency stress and maintain a coherent (or semi-coherent) phase boundary upon (de)lithiation^60^, loss of interface coherency through the generation of dislocations and cracks is commonly observed in microsized particles^13, 16, 18, 61^, which leads to stress relaxation. As will be described below, we also performed control simulations assuming coherent phase boundary and compared the result to experiment to further support that misfit stress is largely relieved in our sample.

As shown in Fig. 5b and also the Supplementary Movie 2, phase-field simulation exhibits dynamic-phase morphology similar to what was observed under *operando* TXM-XANES (Fig. 3). FP phase initially nucleates on (100)/(001) surface at *x = *0 and grows both parallel and perpendicular to the surface. The domain size of the growing FP phase along [100]/[001] tapers at the moving TPJ. The aspect ratio of FP phase, defined by *L*/*R* shown in Fig. 5b, continues to increase with delithiation, suggesting faster growth along [010]. To gain more insights on the growth kinetics, *L* and *R* are plotted against time in a log–log plot in Fig. 5c. They clearly follow different scaling behaviors. The [100]/[001] dimension of FP phase increases with time as *R*(*t*) ∝ *t* ^1/2^. In fact, this parabolic relation is obeyed at not only *x = *0 but all other locations along (100)/(001) surface (Supplementary Fig. 3), suggesting diffusion-limited phase-boundary movement in the [100]/[001] direction. In contrast, the [010] dimension *L*(*t*) exhibits a linear growth behavior after a transient period (*L*(*t*) ∝ *t*), which is not consistent with BDL behavior but agrees with the SRL phase-boundary movement kinetics predicted by the SCB theory^18, 19^. Simulation shows that the TPJ velocity *v*_*L*_ = d*L*/d*t* increases linearly with *ϕ*_low_ below the spinodal point (Fig. 5d). The different time-dependent behaviors of *L*(*t*) and *R*(*t*) indicate that the growth of FP phase is governed by distinct mechanisms along different directions and thus operates in a hybrid mode that has not been observed or discussed before. Here we define the hybrid-mode phase-boundary migration as different segments of the phase boundary following different kinetics (SRL or BDL) in their movement.

Although the system size (200 nm × 400 nm) used in our simulation is smaller than the experimental particle dimensions by one decade, the well-defined scaling characteristics of *L*(*t*) and *R*(*t*) allow simulation results to be reliably extrapolated to larger length and time scales. As shown in Fig. 4a, the extrapolated *R*(*t*) using *ϕ*_low_ = 40 mV shows satisfactory agreement with the experiment considering the uncertainties in parameter values used in simulation. Compared to the estimate based on Eq. 1 (dashed line in Fig. 4a), phase-field simulation predicts a lower growth rate along [100]/[010] because a smaller diffusivity is used and non-uniform delithiation on (100)/(001) surfaces reduces the Li flux along [100]/[001] at *x* = 0. In the [010] direction, the simulated growth rate matches the measurement at *ϕ*_low_ ≈ 40 mV. Compared to the simulation, the experimental *L*(*t*) displays a slowdown in growth speed. This discrepancy may result from a continuous decreasing overpotential along the [100]/[010] surfaces due to polarization caused by bulk or surface electrical resistance instead of the constant overpotential assumed in simulation. However, a more quantitative comparison with the experiment requires the knowledge of overpotential distribution on the surface of the image particle, which could not be obtained in our experiment. Nevertheless, the fact that experiment and simulation can reach good agreement under reasonable parameter values already supports the conclusion that the fast [010] growth of FP phase observed in TXM cannot be explained by diffusion-controlled phase-boundary migration but could be attributed to the SRL mechanism. Therefore, our experiment provides the first direct observation of the predicted SRL phase-boundary migration kinetics in intercalation compounds. Furthermore, we show that the facile ion diffusion assumed in SCB theory is not a necessary condition for observing SRL phase-boundary movement. Even when Li diffusion can no longer be considered as much faster than surface reaction, e.g., due to the presence of anti-site defects or large particle size, a portion of the phase boundary can still travel along particle surface at a constant speed and obey SRL kinetics, whereas the other boundary segments follow BDL kinetics.

Phase-field simulation also shows that phase boundary maintains a stable morphology behind the TPJ when it travels as a constant speed during delithiation (Fig. 5e), which can be well fitted by a parabola, which is the dashed line in Fig. 5e. This behavior implies that a local steady state of Li transport has been established around the TPJ or [010] growth front of FP phase. The parabolic boundary profile is the consequence of BDL phase-boundary movement along [100]/[001]. To see this, suppose that the TPJ reaches a location *x* at time *t*_0_(*x*). After *t* = *t*_0_(*x*), the [100]/[001] size of FP domain at *x* should increase parabolically as *y* (*x*, *t*) = $\lambda \sqrt {t - {t_0}(x)}$, where *λ* is a constant, since the [100]/[001] growth is diffusion-limited. Because the TPJ moves at a constant velocity *v*_L_, *t*_0 =_ *x*/*v*_L_ and *t = L*/*v*. Using these relations we obtain *y* (*x*, *t*) = $\lambda _L^{ - 1{\rm{/}}2}\sqrt {L(t) - x}$, which provides a simple mathematical description of the phase-boundary evolution during the hybrid-mode phase transformation. Although the resolution of TXM phase maps (1 pixel corresponding to ~160 nm) does not permit a quantitative validation of this prediction, the single-phase chemical maps show the similar tapered morphology of FP phase towards the TPJ. The simulated phase-boundary profile differs from the fitted curve near *x* = 0 in Fig. 5e. The reason for the difference is that LFP and FP phases are given the same surface energy in simulation, which requires the phase boundary to form a 90° contact angle with (010) surface and thus deviate from the parabolic trajectory.

Here we consider the potential effects of several factors, i.e., coherency stress, Li-diffusion anisotropy and the mobility of misfit dislocations, on the interpretation of our experimental observation. To examine the possible involvement of coherency stress in regulating phase transformation in the microrod particle, another simulation that assumes fully coherent phase boundary but otherwise employs identical model parameters was carried out. As shown in Supplementary Fig. 4a and b, the simulated FP growth morphology differs significantly from the previous simulation without coherency stress (Fig. 5b) and does not display the tapered shape observed experimentally. Furthermore, the aspect ratio *L*(*t*)/*R*(*t*) of the FP domains obtained from this simulation is close to isotropic and much smaller than the experimental value when extrapolated to *R* = 500 nm (Supplementary Fig. 4c). We also performed a simulation to examine whether the combination of anisotropic Li diffusion and coherency stress can reproduce the experimental observation in the absence of surface reaction on (100)/(001) surfaces. This study is motivated by the theoretical predictions^33, 59^ that tensile strain can significantly reduce the Li-migration barrier in LFP, which may enable a much higher Li diffusivity along [010] to account for the observed FP growth rate. However, when 1D Li diffusivity along [010] is assumed in the simulation, the obtained shape evolution (Supplementary Fig. 5a) and aspect ratio (Supplementary Fig. 5b) of the FP domains again exhibit considerable difference from the TXM observation (Fig. 3). The difference between the simulated and experimental phase morphology should be even more pronounced for smaller Li-diffusion anisotropy (i.e., non-zero [100]/[001] Li diffusivity), where the [100]/[001] dimension of FP phase expands at a higher rate and further lowers the *L*/*R* ratio. These comparisons support that the misfit stress in LFP microrod particles is largely relaxed presumably through loss of interface coherency, and bulk diffusion alone without surface reaction on the (100)/(001) surfaces cannot explain the observed phase-boundary morphology.

Although our analysis of the TMX data suggest that the [100]/[001] Li diffusivity *D*_[100]/[001]_ is at least comparable to the [010] diffusivity *D*_[010]_ in the microrod particles, the possibility of *D*_[100]/[001]_ being much larger than *D*_[010]_ needs to be examined. A simulation was performed with the assumption of 1D Li diffusivity in the [100]/[001] direction (i.e., no [010] Li transport) and otherwise same parameters as in the previous simulation assuming 2D Li diffusivity and no coherency stress. The result (Supplementary Fig. 6) shows that the obtained phase-boundary profile is much less smooth than in the simulation with 2D Li diffusion. An abrupt change in the [100]/[001] dimension *R* of FP phase is visible at the location where the applied overpotential has a stepwise change, corresponding to the boundary between the (100)/(001) surface regions with and without electrical contact. As shown in Supplementary Fig. 6, due to the lack of Li diffusion along [010], the aspect ratio *L*/*R* of FP domains, in which *R* is measured in the region without electrical contact, is significantly larger than in the simulation with *D*_[010]_ = *D*_[100]/[001]_ at the early stage of delithiation. However, it only increase slightly when phase transformation proceeds (Supplementary Fig. 6c), which is distinct from the experimental observation (Fig. 3). The better agreement between the experiment and the simulation assuming 2D Li diffusivity further verifies that Li should have similar diffusivity values along [100]/[001] and [010] in our sample.

In addition to the SRL and BDL phase-boundary movement, it is also generally possible that the migration of incoherent phase boundary could be controlled by the mobility of misfit dislocations present on the interface. However, the observation that the [100]/[001] growth of FP phase can be well described by the BDL kinetics using the experimentally measured Li diffusivity value suggests that the motion of misfit dislocations is not a likely rate-limiting step for phase-boundary movement in our sample. This is consistent with a recent in situ TEM observation of microsized LFP particles^16^, which finds misfit dislocations on (010) phase boundary to be quite mobile.

### Different phase-transformation mechanisms in LiFePO_4_

**Fig. 6:**
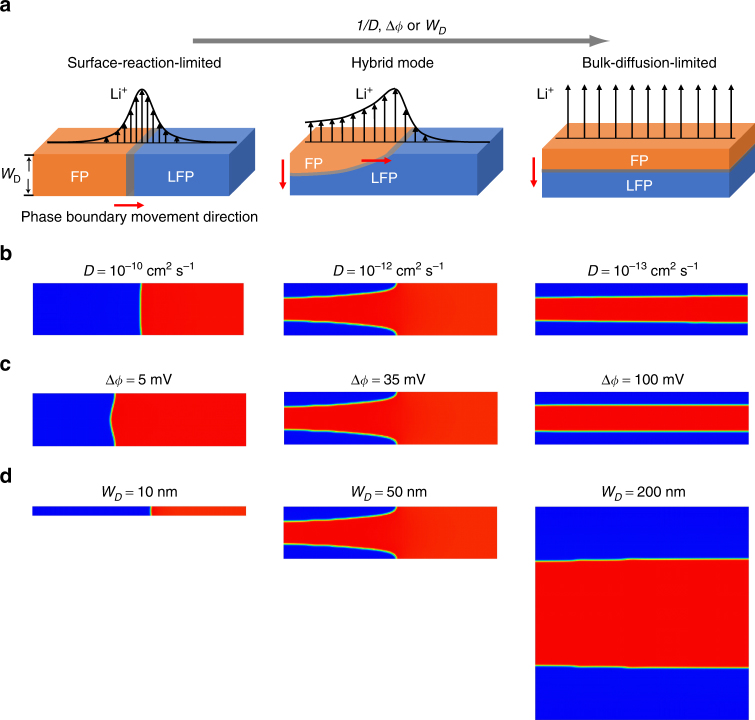
Different phase-transformation mechanisms in LiFePO_4_. **a** Schematics of three kinetic regimes of phase-boundary migration in intercalation compounds, from left to right: SRL, hybrid, and BDL mode. Phase-field simulations illustrate the transition from SRL to hybrid and then BDL-boundary movement in LiFePO_4_ upon **b**, decreasing Li diffusivity *D*, **c** increasing overpotential Δ*ϕ* and **d** increasing particle thickness along the main Li intercalation direction *W*_*D*_. An exchange current density of *j*_0_ = 1 A m^–2^ and 2D Li diffusivity are used in all simulations. Δ*ϕ* = 35 mV and *W*_*D*_ = 50 nm for simulations shown in **b**, *D* = 10^−12 ^cm^2^ and *W*_*D*_ = 50 nm for simulations in **c**, and *D* = 10^−12 ^cm^2 ^s^−1^ and Δ*ϕ* = 35 mV for simulations in **d**

The combined TXM-XANES experiment and phase-field simulation not only confirm the existence of SRL phase-boundary migration in LFP, but also lead to a surprising finding that two different boundary migration modes (SRL and BDL) can operate simultaneously in LFP. The SCB theory^19^ predicts the SRL boundary movement when Li bulk diffusion is much more facile than Li insertion/extraction across the electrode/electrolyte interface. In this limit, phase boundary extends from surface to surface along Li intercalation direction and moves in the direction orthogonal to surface flux, as illustrated in Fig. 6a. Expanding the prediction of SCB theory, we suggest that there are three general kinetic regimes of phase-boundary movement in intercalation compounds when interface reaction (or ion hopping across phase boundary) is not a rate-limiting step. They include the pure SRL and BDL regimes as well as an intermediate hybrid regime that entails the features of both SRL and BDL dynamics. Schematics of phase-boundary movement in relation to Li (de)intercalation in the three regimes are illustrated in Fig. 6a. Which kinetic regime the phase-transformation process falls into depends on the relative rates of Li bulk diffusion vs. surface reaction, which are modulated by multiple materials and electrochemical parameters. In Fig. 6b, we demonstrate through phase-field simulation that FP phase growth will transition from pure SRL to hybrid and then pure BDL behavior with increasingly small Li diffusivity *D*. In addition, increasing the applied overpotential Δ*ϕ* will also promote BDL-boundary movement (Fig. 6c) because of the non-linear dependence of surface flux on Δ*ϕ* in the Butler–Volmer reaction kinetics. Enlarging particle size *W*_*D*_ along the Li intercalation direction has a similar effect since it increases the Li-diffusion length (Fig. 6d). The different phase-boundary evolution behavior in the three regimes also causes the ion intercalation kinetics to be qualitatively different. Upon (dis)charge under a constant overpotential, the state of (dis)charge of electrode particles varies with time as *t*, *t* ^3/2^, or *t* ^1/2^ in the SRL, hybrid, or BDL regime, respectively. The existence of multiple kinetic modes thus presents additional complexity for accurately predicting the rate performance of intercalation compounds that undergo electrochemically driven phase transformations.

In a previous work, Weichert et al.^27^ examined the chemical delithiation of LFP single crystals subject to very large overpotentials, and found that (001) crystal surface was uniformly delithiated and the FP layer thickness increased with time parabolically. This study provided a direct observation of phase transformation in the BDL regime. In another notable in situ TEM study^16^, Zhu et al. investigated the lithiation process in microsized FP particles in an all solid electrochemical cell setup. Upon applying a large voltage, a thin LFP layer formed on (010) surface and thickened uniformly along [010] within the imaging region, which is also consistent with BDL growth. Interestingly, they observed in one particle a phase boundary that is slightly tilted from (010) plane, which might suggest the hybrid growth mode. However, this possibility cannot be confirmed since only a small section of phase boundary was imaged in TEM. We note that a recent in situ scanning TEM study of the hydrogenation of Pd nanocrystals reveals a phase-growth morphology that resembles the hybrid-mode phase transformation, where the faster phase-boundary motion along particle surface and the tapered growth front are visible (Fig. 1 in ref. ^62^). We propose that the hybrid phase-boundary migration is a general mechanism for intercalation compounds.

## Discussion

In summary, the phase-transformation process in single-crystalline LFP microrods has been investigated by *operando* TXM-XANES imaging and phase-field simulation. TXM chemical mapping data reveal 2D Li-diffusion behavior in hydrothermally synthesized LFP as against the theoretically predicted 1D Li diffusivity for perfect olivine structure. This finding supports that the effect of anti-site defects on reducing Li-diffusion anisotropy in LFP is universal and independent of synthesis techniques. We obtained direct evidence that Li ions can be intercalated through the (100)/(010) surfaces, contradicting a common belief that only the (010) surface of LFP is electrochemically active. The TXM-XANES study also presents the first experimental confirmation of the previously hypothesized SRL phase-boundary migration in LFP. On the basis of the analysis of the TXM data and phase-field modeling, we propose that a new hybrid mode of phase transformation provides the most probable explanation of the observed phase-growth morphology and kinetics, in which the phase-boundary migration is controlled by surface reaction or Li diffusion in different crystallographic directions. Accordingly, three distinct kinetic regimes (SRL, hybrid, and BDL) of phase transformations are expected to exist in LFP and potentially many other intercalation compounds. Our work stresses that the interplay between surface reaction and bulk diffusion kinetics could produce a variety of phase-transformation kinetics in ion-insertion materials that need to be carefully considered in the design and modeling of high-performance battery electrodes.

## Methods

### Synthesis of the LiFePO_4_ microrods

To prepare the LFP microrods, 6 mmol of ammonium dihydrogen phosphate (NH_4_H_2_PO_4_), lithium hydroxide monohydrate (LiOH·H_2_O), and nitrilotriacetic acid [N(CH_2_CO_2_H)_3_] were weighed out and transferred into a glass jar, to which 60 mL of deionized water was then added and the solution was stirred for 15 min. Then 1−2 mL of concentrated NH_3_·H_2_O were micropipetted into the solution until a pH of 9 was attained. Overall, 6 mmol of iron(II) sulfate heptahydrate (FeSO_4_·7H_2_O) was then added to the solution and the solution was stirred for another 15 min. The resulting suspension was transferred to a Teflon reactor in a stainless steel autoclave that was heated in an oven at 200 °C for 24 h and then allowed to cool down naturally. The greenish precipitate was collected by centrifugation and washed with deionized water and ethanol several times before drying in an oven at 60 °C.

### Material characterizations

SEM images were acquired using a LEO 55 VP field emission scanning electron microscope at a working voltage of 5 kV. TEM images and electron diffraction (ED) patterns were acquired using a FEI Philips FM200 transmission electron microscope (200 kV). Synchrotron PXRD was performed at beamline 11-ID-B (*λ* = 0.2128 Å) at the Advanced Photon Source, Argonne National Laboratory. The sample was prepared by loading fine powders of LFP into a polyimide capillary (Kapton, O.D. 0.0435″). The raw images were transformed to powder diffraction patterns using the FIT2D program. Rietveld refinements of crystal structure were performed using FullProf program under two different models: (1) LFP with no anti-site defects; (2) LFP with anti-site defects. First, scale factor and 50 background points were refined. The background was a linear interpolation between the 50 points using cubic splines. Then unit cell parameters were refined, followed by profile parameters. After initial refinement converged, atomic positions (coordinates) were released and refined in the order of decreasing atomic number. Subsequently, atomic displacement parameters (*U*_iso_) were released and refined from Fe, P to O. Finally, site occupancy factors were refined.

### Electrochemical characterization

Electrochemical discharge and charge tests were performed on composite electrodes pasted on aluminum foils, which were prepared from slurries containing 70 wt% active material, 20 wt% conductive carbon black, and 10 wt% PVDF binder using NMP as the solvent. CR2032-type coin cells were assembled in an argon-filled glovebox, using Li metal as the counter electrode, 1 M LiPF_6_ in EC/DMC (1/1 by volume, BASF) as the electrolyte, and Celgard 2300 membrane as the separator. GITT measurements were carried out to determine the effective Li diffusivity at *x = *0.01 for Li_1−*x*_FePO_4_. The cell was allowed to relax for 24 h or until d*E*/d*t < *0.1 mV h^−1^ after every 12 min charging at a current of 1/20 *C* (1 *C* = 170 mAh g^−1^). The diffusivity was calculated using

3 $${D_{{\rm{GITT}}}} = \frac{4}{\pi }\left( {\frac{{I{V_{\rm{M}}}}}{{{Z_{\rm{A}}}FS}}} \right){\left[ {\frac{{{\rm{d}}E\left( x \right){\rm{/d}}x}}{{\left( {{\rm{d}}E\left( t \right){\rm{/d}}\sqrt t } \right)}}} \right]^2}.$$

*I* (A) is the applied current, *V*_M_ (cm^3 ^mol^−1^) is the molar volume of the electrode material, *z*_A_ is the charge number of the electroactive species (*z*_A = _1 for Li^+^), *F* is the Faraday constant, and *S* (cm^2^) is the LFP-electrolyte contact area. *S* is calculated from the side surface area of the microrods, which have an average width of ~5 μm as estimated from SEM images.

### *Operando* TXM-XANES

The *operando* TXM-XANES experiments were performed using the full-field transmission X-ray microscope (FFTXM) at beamlineX8c, National Synchrotron Light Source (NSLS), Brookhaven National Laboratory (BNL), using a perforated 2032-type coin cell with holes on both sides sealed by Kapton tapes. The *operando* measurements were performed on diluted electrodes made of 50 wt% LFP active material, 30 wt% carbon black, and 20 wt% PVDF binder to minimize overlapping between particles. Carbon microfiber papers (~110 μm thickness, Fuel Cell Earth LLC) are used as current collectors because they are highly porous and quite transparent to hard X-rays. The cell was put into a custom-built holder mounted on a motorized *X*, *Y*, *Z*, *θ* stage and aligned to allow the X-ray beam to transmit through. A field-of-view of 40 × 40  μm^2^ with a 2048 × 2048 CCD camera was used. The cell was continuously charged in constant-current (1/5 *C*) or constant-voltage mode and absorption-contrast TXM images (X-ray transmitted through the sample) and reference background images (X-ray passing through air) were collected in sequences. To track the electrochemical reaction, a full series of TXM images were collected at each state of charge. Each TXM image series was collected by scanning across the Fe *K*-edge (7112 eV) from 7091 to 7285 eV, with a step size of 2 eV, one image at each energy, which contains 256 × 256 XANES spectra when 8 × 8 binned camera binning was used. The exposure time for each image was 2 s. Each chemical-phase map took ~7 min to finish. After collection each set of data, the area of study was allowed to rest for ~14 min (not exposed to X-rays) to minimize any potential impact induced by the X-ray beam while the battery was continuously charging. The output pixel size is ~160 nm (camera binning 8).

The XANES spectrum at each pixel was normalized using an established method^37^ and then fitted with the linear combination of standard reference spectra collected from Fe^3+^PO_4_ and LiFe^2+^PO_4_ powders sealed between two pieces Kapton otherwise under the same conditions using TXM. The spectrum fitting was carried out by minimizing the *R*-value (a measure of misfit) for each spectrum at each pixel, which is defined as:

4 $$R = {\mathop {\sum}\limits_{Ei}^{Ef} {\left( {dataE - refE} \right)} ^2}{\rm{/}}\mathop {\sum}\limits_{Ei}^{Ef} {data{E^2}} ,$$

where *Ei* is 7091 eV, *Ef* is 7285 eV, *dataE* is the normalized spectrum at each pixel for the given energy *E*, and *refE* is the possible fitting reference value that is a linear combination of X-ray attenuation of LFP and FP. *R* values were minimized at each pixel to find the best-matched phase combination of different Fe oxidation states so that red (Fe^3+^PO_4_) and green (LiFe^2+^PO_4_) colors can be assigned accordingly to generate the two-phase maps. *R-*value filter (misfit filter) was applied to the resulting phase map to give the most accurate chemical-phase information. *R* is typically smaller than 0.05. Single-phase maps were generated ImageJ using information included in the two-phase maps. We split the RGB channels and applied “jet” lookup-table (LUT) to the Green-channel image, where the color-scale indicates phase fraction of the FP phase.

### Phase-field modeling

The delithiation process in LFP is simulated with a phase-field model^20^. In the model, the site occupancy fraction of lithium $c(r ^{\!\!\!\!\rightarrow})$ is used as the order parameter to distinguish between LFP (*c* = 1) and FP (*c* = 0) phases. The time evolution of $c(r ^{\!\!\!\!\rightarrow})$ is governed by the Cahn–Hilliard equation^63,64^

5 $$\frac{{\partial c}}{{\partial t}} = \nabla \cdot \left[ {\frac{{D{V_{\rm{m}}}}}{{RT}}c\left( {1 - c} \right)\nabla \left( {\frac{{\partial {f_{{\rm{chem}}}}\left( c \right)}}{{\partial c}} - \kappa {\nabla ^2}c} \right)} \right],$$

which describes both diffusion and phase-boundary migration processes in phase-separating systems. Here *D* is diffusion coefficient matrix, *V*_m_ is the molar volume and *R* is gas constant. The homogeneous chemical free-energy density *f*_chem_ is given a regular solution expression with the regular solution coefficient *Ω* set as 12 kJ mol^−1^ for LFP^65^. The gradient coefficient *κ* is given a value of 1.68 × 10^−12^ J cm^−1^, which produces an interface energy of 0.072 J m^−2^ that is the average value of the (100), (010), and (001) interface energies from first-principles calculations^21^. In the simulation that assumes coherent phase boundary (Supplementary Fig. 4), the stress-equilibrium equation is also solved using the same linear elasticity formulation and elastic parameters as in ref. ^20^

As the phase distribution in the LFP particle along the X-ray beam direction cannot be discerned by the 2D TMX technique, 2D simulation is performed to reduce computation cost. A system size of *W*_[010]_ × *W*_[100]/[001]_ = 400 nm × 200 nm is chosen. In accordance with the observation that the particle is mainly delithiated through (100)/(001) surface and (010) surface is inactive in Li deintercalation due to poor contact with the conductive network, a zero Li flux boundary condition is imposed on (010) surface. Li flux on (100)/(001) surface is described by the Butler–Volmer equation:

6 $${j_{{\rm{Li}}}} = \frac{{{V_{\rm{m}}}}}{F}{j_0}\left[ {\exp \left( {\frac{{{\alpha _{\rm{c}}}\left( {\mu _{{\rm{Li}}}^{{\rm{electrolyte}}} - \mu _{{\rm{Li}}}^{{\rm{surf}}}} \right)}}{{RT/{V_{\rm{m}}}}}} \right) - \exp \left( { - \frac{{{\alpha _{\rm{a}}}\left( {\mu _{{\rm{Li}}}^{{\rm{electrolyte}}} - \mu _{{\rm{Li}}}^{{\rm{surf}}}} \right)}}{{RT/{V_{\rm{m}}}}}} \right)} \right],$$

where *j*_0_ is the exchange current density, $\mu _{{\rm{Li}}}^{{\rm{electrolyte}}}$ and $\mu _{{\rm{Li}}}^{{\rm{surf}}}$ are the Li chemical potential in electrolyte and on particle surface, respectively, *F* is the Faraday constant, and the charge-transfer coefficients are set to *α*_a_ = *α*_c_ = 0.5. $\mu _{{\rm{Li}}}^{{\rm{electrolyte}}}$ is related to the applied overpotential, which is defined as $\Delta \phi = ( {\mu _{{\rm{Li}}}^{{\rm{eq}}} - \mu _{{\rm{Li}}}^{{\rm{electrolyte}}}} ){\rm{/}}F$, where $\mu _{{\rm{Li}}}^{{\rm{eq}}}$ is the Li chemical potential at LFP/FP two-phase equilibrium.

### Data availability

The data that support the findings of this study are available from the corresponding author upon request.

## Electronic supplementary material

Supplementary Information

Description of Additional Supplementary Files

Supplementary Movie 1

Supplementary Movie 2
