# Supplementary material for: Two-dimensional lithium diffusion behavior and probable hybrid phase transformation kinetics in olivine lithium iron phosphate
Source: Nat Commun. 2017 Oct 30;8:1194. doi: 10.1038/s41467-017-01315-8 (PMC5662729; doi:10.1038/s41467-017-01315-8)
Supplement: Supplementary file 1 — Supplementary Information [file 41467_2017_1315_MOESM1_ESM.pdf]

Supplementary Figures

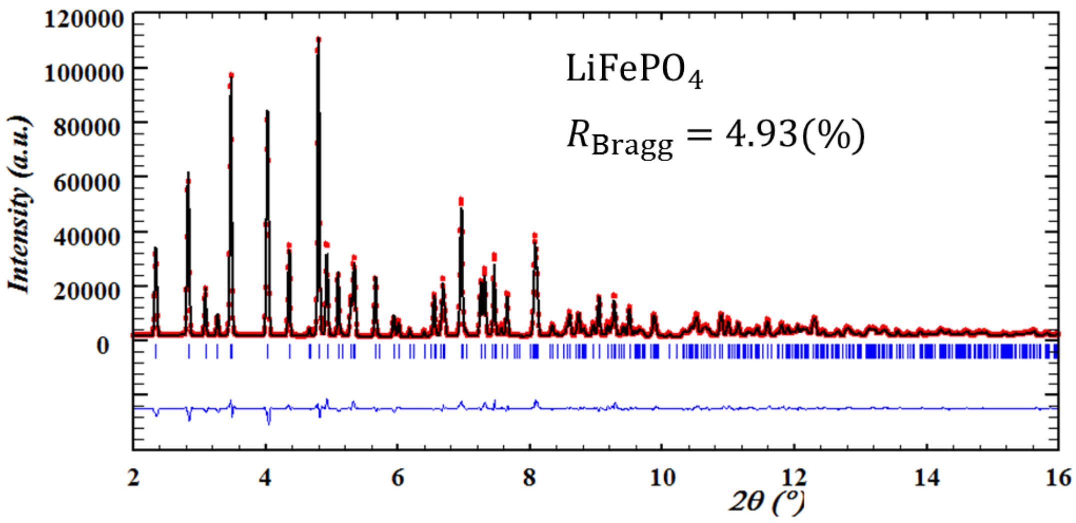

Supplementary Figure 1 | High-resolution PXRD of the $LiFePO_4$ microrods and the Rietveld-refined pattern using $LiFePO_4$ with no anti-site defects. Diffraction data was collected at beamline 11-ID-B (λ = 0.2128 Å) at the Advanced Photon Source, Argonne National Laboratory.

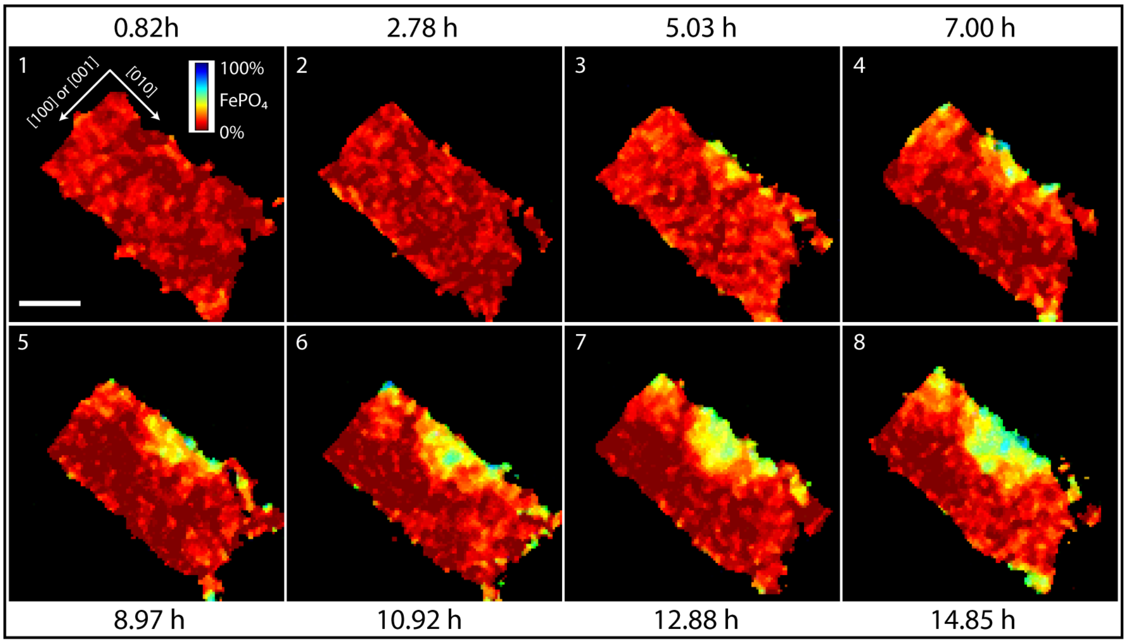

Supplementary Figure 2 | *Operando* TXM visualization of delithiation of another single-crystal $LiFePO_4$ microrod. 2D o*perando* $FePO_4$ single-phase chemical maps (115 × 115 pixels) taken at different states of charge show the evolution of the $FePO_4$ phase. The "jet" color-scale corresponds to the fraction of the FP phase (red, 0% $FePO_4$; blue, 100% $FePO_4$). The long-axis of the $LiFePO_4$ microrod is along [010] and its short-axis is along [100] or [001]. Map 1 was collected at 3.48 V vs. $E(Li^+/Li)$ while Maps 2 to 8 were collected at 3.52 V vs. $E(Li^+/Li)$. These chemical maps clearly show that the delithiation initiated on (100)/(001) instead of (010) particle surfaces.

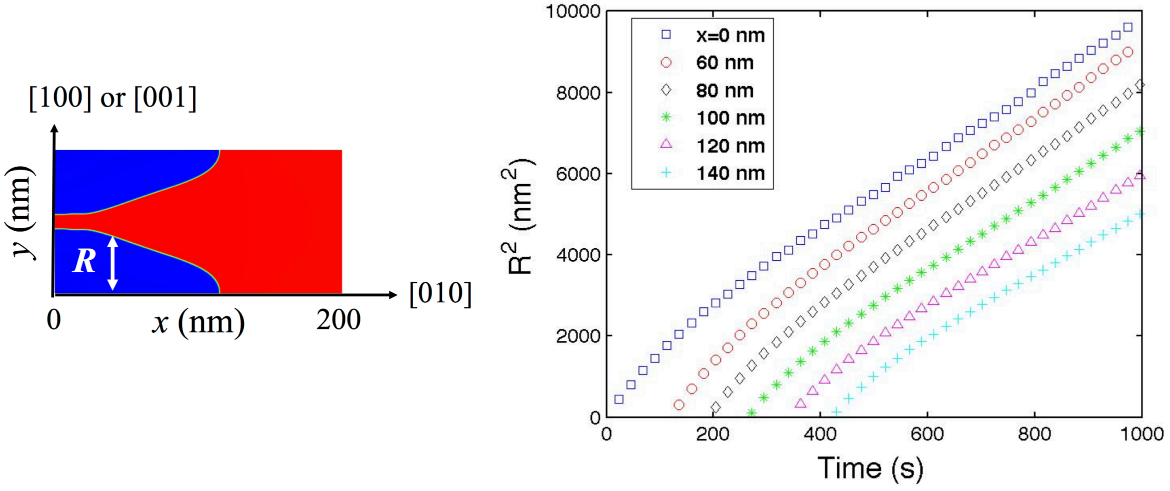

Supplementary Figure 3 | [100]/[001] dimension of FP phase *R* vs. time measured at different locations along the [010] axis. After an initial period, R^2 exhibits linear dependence on time at all the locations, suggesting that diffusion-limited growth in [100]/[001] is followed everywhere along the phase boundary.

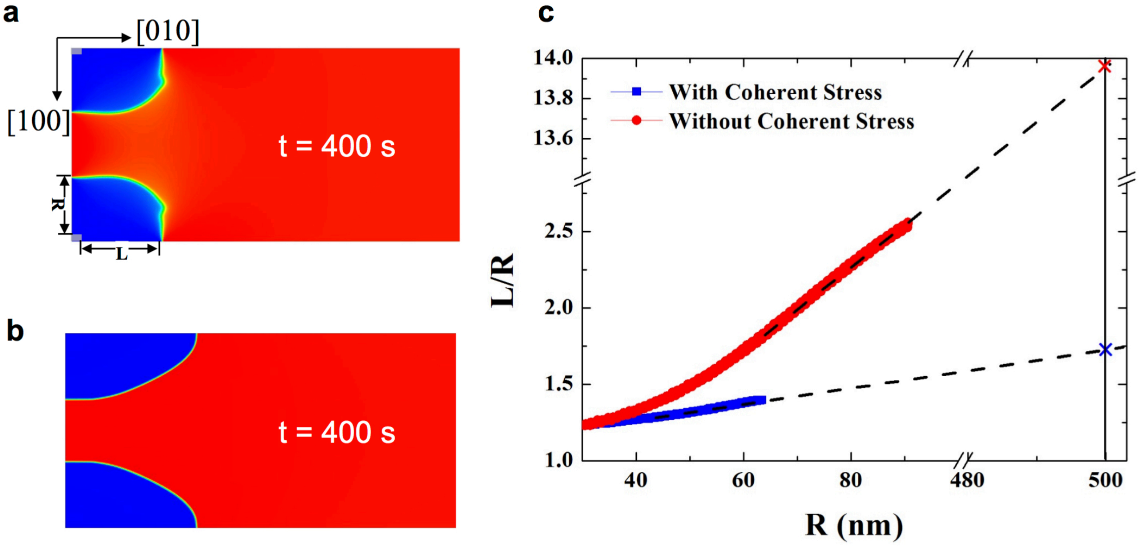

Supplementary Figure 4 | Comparison of the growth morphology of FP phase upon delithiation with and without coherency stress present in the particle. **a** and **b**, The FP phase morphology at $t = 400$ s from simulations assuming coherency stress and stress-free growth, respectively, in which the same parameters as in the simulation shown in Figure 5 are used. **c**, The aspect ratio L/R of the FP phase vs its [100]/[001] dimension R from simulations with and without coherency stress. Dashed lines represent the extrapolation of the simulation results to the experimental domain size $R \approx 500$ nm.

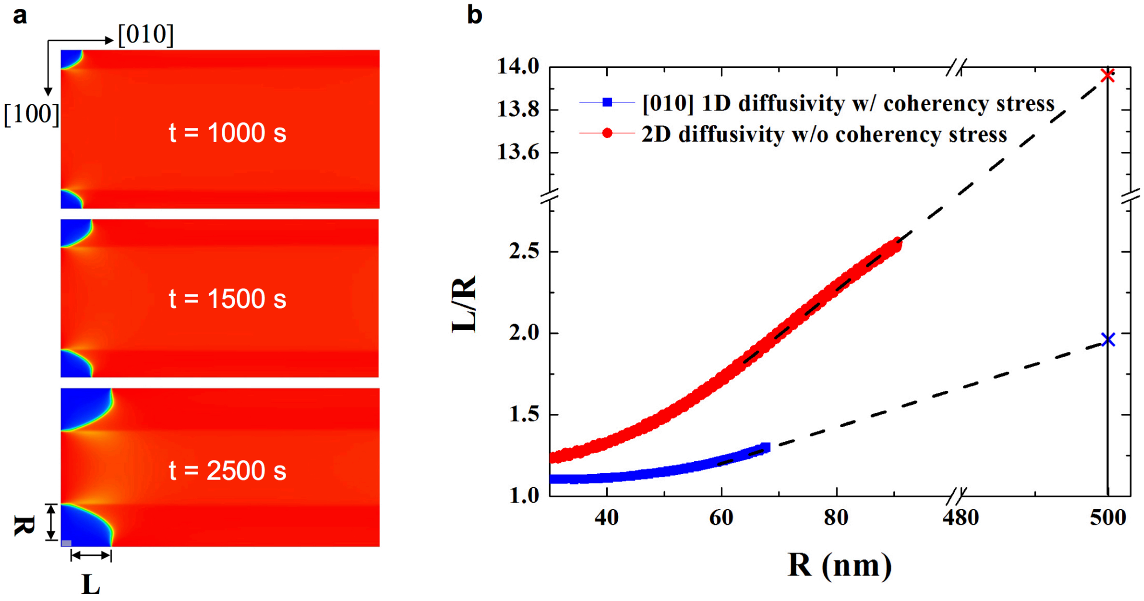

Supplementary Figure 5 | Delithiation simulation assuming 1D Li diffusion along the [010] axis and the presence of coherency stress. Because the (100)/(001) surfaces can no longer deintercalate Li in this case, the left (010) boundary of the computational domain is assumed to be electrochemically active with an applied overpotential of 35 mV. **a**, Snapshots of the FP morphology at different simulation times. **b**, Evolution of the aspect ratio *L*/*R* of the FP phase during its growth (blue) compared with the result from the simulation with 2D diffusivity and without coherency stress (red).

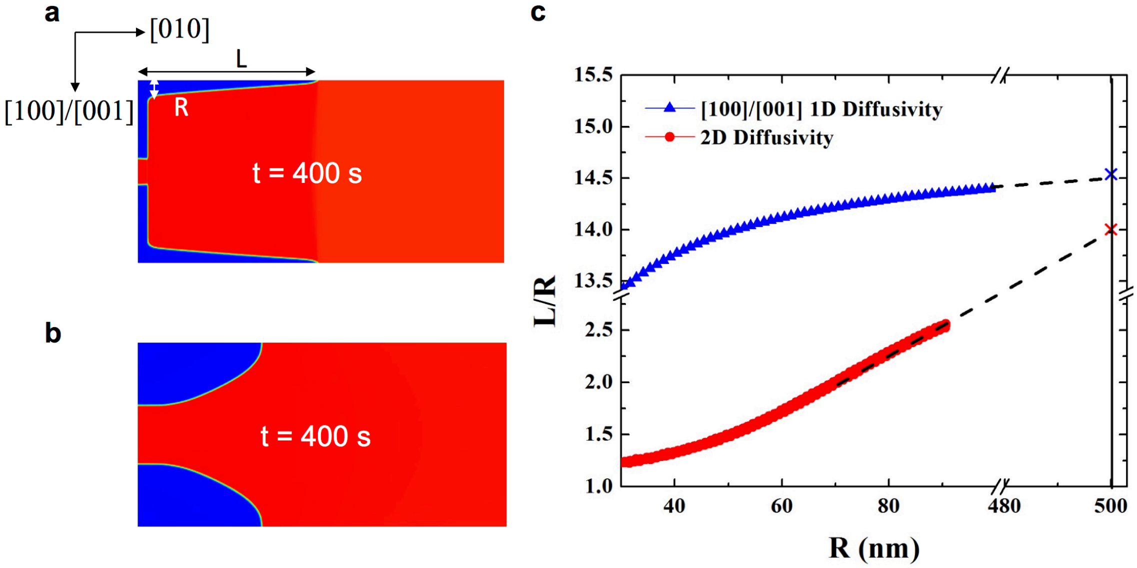

Supplementary Figure 6 | Comparison of the growth morphology of FP phase upon delithiation with [100]/[001] 1D Li diffusivity and 2D Li diffusivity ($D_{[100]/[001]} = D_{[010]}$). a and **b**, The FP phase morphology at t = 400 s from simulations assuming [100]/[001] 1D and 2D Li diffusivity, respectively, in which the same parameters as in the simulation shown in Figure 5 are used. **c**, The aspect ratio *L*/*R* of the FP phase vs its [100]/[001] dimension *R* from simulations with [100]/[001] 1D and 2D Li diffusivity. *R* in the simulation with 1D Li diffusivity is measured at the location where the overpotential has a stepwise change on the (100)/(001) surface.

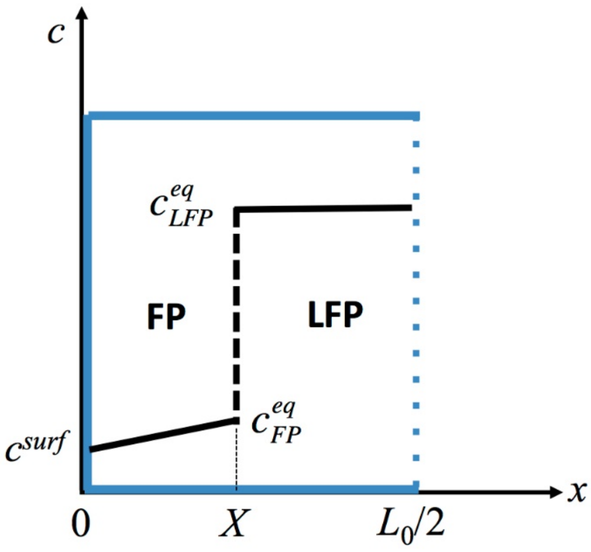

Supplementary Figure 7 | Schematics of Li concentration distribution in a $LiFePO_4$ particle during delithiation. Only half of the particle is shown. Li concentration is assumed to be symmetric profile in the other half.

Supplementary Notes

Kinetic model of diffusion-limited phase transformation in $LiFePO_4$

Here we derive a simple model of phase transformation kinetics in LFP assuming that phase boundary migration is bulk-diffusion limited. Consider that delithiation proceeds symmetrically from a LFP plate-like particle of length L_0 as illustrated in Supplementary Fig. 7. The advancement of the phase boundary location X is governed by the Stefan condition:

$$\left(c_{LFP}^{pb} - c_{FP}^{pb}\right)\frac{dX}{dt} = J_{FP}^{pb} - J_{LFP}^{pb} \tag{1}$$

where c_{LFP}^{pb} and c_{FP}^{pb} are the Li concentrations of LFP and FP phases at the phase boundary, respectively, J_{LFP}^{pb} is the Li flux entering the phase boundary on the LFP side, and J_{FP}^{pb} is the flux leaving the boundary on the FP side. For diffusion-limited growth, FP and LFP phases are in local equilibrium with each other at the phase boundary, i.e., they have the equilibrium Li concentrations at two-phase coexistence, $c_{LFP}^{pb} = c_{LFP}^{eq}$ and $c_{FP}^{pb} = c_{FP}^{eq}$. As an upper limit estimate of the phase boundary speed, LFP phase is assumed to have a uniform Li concentration at c_{LFP}^{eq}, and so $J_{LFP}^{pb} = 0$. Because Li has small solubility in FP phase, Li diffusion in FP phase can be approximated as quasi-steady state, and so the Li flux leaving the phase boundary is evaluated as:

$$J_{FP}^{pb} = D\frac{dc}{dx} = D\frac{c_{FP}^{eq} - c^{surf}}{X} \tag{2}$$

Supplementary Equation 1 thus becomes

$$\left(c_{LFP}^{eq} - c_{FP}^{eq}\right)\frac{dX}{dt} = D\frac{c_{FP}^{eq} - c^{surf}}{X} \tag{3}$$

where c^{surf} is the Li concentration at surface. When the particle is subject to a constant overpotential, c^{surf} is kept at a constant level and the above equation can be integrated to obtain Eq. 1 in the main text, i.e.

$$X(t) = \sqrt{\frac{c_{FP}^{eq} - c^{surf}}{c_{LFP}^{eq} - c_{FP}^{eq}} 2Dt}$$

If the particle is subject to constant current delithation, J_{FP}^{pb} is required to be also constant to sustain the kinetics, and its value is related to the maximal charge time t_C at a given C rate (i.e. t_C = 3600 s/C) as

$$J_{FP}^{pb} = (c_{LFP}^{eq} - c_{FP}^{eq})L_0 / (2t_C) \tag{4}$$

Additionally, the relation between the state of charge (SOC) and the phase boundary location is:

$$SOC = 2X / L_0 \tag{5}$$

Using Supplementary Equation 2, 4 and 5, we derive an expression for predicting how the surface Li concentration c^{surf} varies with SOC upon delithation

$$c^{surf} = c_{FP}^{eq} - \frac{(c_{LFP}^{eq} - c_{FP}^{eq})L_0^2}{4Dt_C} \times \mathrm{SOC} \tag{6}$$

Because c^{surf} cannot drop below 0, the maximal obtainable capacity at a given C rate is

$$(\mathrm{SOC})_{\max} = \frac{4c_{FP}^{eq}Dt_C}{(c_{LFP}^{eq} - c_{FP}^{eq})L_0^2} \tag{7}$$

Supplementary Movie 1 | Time sequence of operando TXM images of a $LiFePO_4$ microrod particle upon delithiation

Supplementary Movie 2 | Phase-field simulation of $FePO_4$ phase growth upon delithiation (red - $LiFePO_4$, blue - $FePO_4$)
