# Supplementary material for: Two-dimensional lithium diffusion behavior and probable hybrid phase transformation kinetics in olivine lithium iron phosphate
Source: Nat Commun. 2017 Oct 30;8:1194. doi: 10.1038/s41467-017-01315-8 (PMC5662729; doi:10.1038/s41467-017-01315-8)
Supplement: Supplementary file 2 — Description of Additional Supplementary Files [file 41467_2017_1315_MOESM2_ESM.pdf]

Description of Additional Supplementary Files

File Name: Supplementary Movie 1
Description: Time sequence of operando TXM images of a $LiFePO_4$ microrod particle upon delithiation

File Name: Supplementary Movie 2
Description: Phase-field simulation of $FePO_4$ phase growth upon delithiation (red - $LiFePO_4$, blue - $FePO_4$)
